# Chronic sucralose consumption induces elevation of serum insulin in young healthy adults: a randomized, double blind, controlled trial

**DOI:** 10.1186/s12937-020-00549-5

**Published:** 2020-04-13

**Authors:** Nallely Bueno-Hernández, Marcela Esquivel-Velázquez, Raúl Alcántara-Suárez, Angélica Y. Gómez-Arauz, Aranza J. Espinosa-Flores, Karen L. de León-Barrera, Viridiana M. Mendoza-Martínez, Gabriela A. Sánchez Medina, Mireya León-Hernández, Alejandra Ruiz-Barranco, Galileo Escobedo, Guillermo Meléndez

**Affiliations:** 1 Laboratory of Proteomics and Metabolomics, Research Division, General Hospital of Mexico “Dr. Eduardo Liceaga”, Mexico City, Mexico; 2 Clinical Nutrition Division, General Hospital of Mexico Dr. Eduardo Liceaga, Mexico City, Mexico; 3 Division of Clinical Pharmacology Research Division, General Hospital of Mexico “Dr. Eduardo Liceaga”, Mexico City, Mexico; 4 Clinic of Medical and Nutritional Trials (MENTRIALS), Mexico City, Mexico

**Keywords:** Non-nutritive sweeteners, Sucralose, Insulin, Glucose, Placebo-controlled trial

## Abstract

**Background:**

Non-nutritive sweeteners (NNS) are widely consumed by humans due to their apparent innocuity, especially sucralose. However, several studies link sucralose consumption to weight gain and metabolic derangements, although data are still contradictory.

**Objective:**

To determine the effect of acute and chronic consumption of sucralose on insulin and glucose profiles in young healthy adults.

**Material and methods:**

This was a randomized, parallel, double-blind, placebo-controlled trial conducted in healthy young adults from 18 to 35 years old, without insulin resistance. A hundred thirty seven participants were randomized into three groups: a) volunteers receiving 48 mg sucralose, b) volunteers receiving 96 mg sucralose, and c) controls receiving water as placebo. All participants underwent a 3-h oral glucose tolerance test (OGTT) preceded by consuming sucralose or placebo 15 min before glucose load, at two time points: week zero (Wk0) and week ten (Wk10). Serum insulin and glucose were measured every 15 min during both OGTTs.

**Results:**

Compared to Wk0, consumption of sucralose for 10 weeks provoked 1) increased insulin concentrations at 0 min (7.5 ± 3.4 vs 8.8 ± 4.1 μIU/mL; *p* = 0.01), 30 min (91.3 ± 56.2 vs 110.1 ± 49.4 μIU/mL; *p* = 0.05), 105 min (47.7 ± 24.4 vs 64.3 ± 48.2 μIU/mL; *p* = 0.04) and 120 min (44.8 ± 22.1 vs 63.1 ± 47.8 μIU/mL; p = 0.01) in the 48 mg sucralose group; 2) increased blood glucose at − 15 min (87.9 ± 4.6 vs 91.4 ± 5.4 mg/dL; *p* = 0.003), 0 min (88.7 ± 4 vs 91.3 ± 6 mg/dL; *p* = 0.04) and 120 min (95.2 ± 23.7 vs 106.9 ± 19.5 mg/dL; *p* = 0.009) in the 48 mg sucralose group; 3) increased area under the curve (AUC) of insulin in both 48 and 96 mg sucralose groups (9262 vs 11,398; *p* = 0.02 and 6962 vs 8394; *p* = 0.12, respectively); and 4) reduced Matsuda index in the 48 mg sucralose group (6.04 ± 3.19 vs 4.86 ± 2.13; *p* = 0.01).

**Conclusions:**

These data show that chronic consumption of sucralose can affect insulin and glucose responses in non-insulin resistant healthy young adults with normal body mass index (between 18.5 and 24.9 kg/m^2^), however, the effects are not consistent with dose; further research is required.

**Clinical trial registry:**

NCT03703141.

## Background

In the past 40 years, the prevalence of overweight and obesity has increased worldwide to the extent of becoming an epidemic [1]. Sugar overconsumption is widely recognized as a contributing dietary factor to obesity [2]. Consequently, the food industry has developed alternatives to reduce the amount of sugar in food and beverages by replacing it with non-nutritive sweeteners (NNS). NNS can be either artificial or natural and their ingestion provide very low or no calories at all [3]. This is the main reason why NNS are now contained in several foods and drinks that are consumed by million people around the globe [4, 5].

Despite NNS consumption is considered safe for humans [6–8], recent studies have shown that short or long-term use of NNS might be related to metabolic alterations, especially in glucose and insulin homoeostasis [9]; however, evidence is still inconclusive [9–11]. In this regard, NNS consumption has been associated with unbalance of the intestinal microbiota that in turn has been shown to cause metabolic disturbances [12, 13]. However, emerging evidence in obese individuals [10] and mice [11] has now suggested that NNS ingestion is directly associated with altered insulin response during oral glucose tolerance test (OGTT), especially with sucralose. In contrast, other studies have found contradictory results, where sucralose consumption had no effect on insulin or glucose responses during OGTT [14–16]. Although multiple mechanisms including gut microbiota dysbiosis and orosensory stimulation may explain these apparently contradictory results [11, 17, 18], it is still unknown whether frequent sucralose consumption can directly disrupt the metabolic homeostasis in humans.

Thus, the aim of this study was to investigate whether acute or chronic sucralose ingestion produces insulin or glucose alterations in healthy young individuals that daily consume 48 mg or 96 mg sucralose for 10 weeks, a sucralose amount equivalent to one or two diet sodas, respectively.

## Methods

### Trial design

This was a randomized, parallel, double-blind, placebo-controlled trial in young healthy adults, conducted in Mexico City. It met the CONSORT criteria as recommended elsewhere [19].

The study was approved by the Ethics and Clinical Research committees of the General Hospital of Mexico Dr. Eduardo Liceaga (Approval No: DI/16/301/03/022) and followed the principles of the Declaration of Helsinki.

The study was conducted at the Laboratory of Proteomics and Metabolomics, Research Division at the General Hospital of Mexico Dr. Eduardo Liceaga. This trial was registered at clinicaltrials.gov (identifier code: NCT03703141).

### Selection of participants

Healthy volunteers from both sexes, aging 18–35 years were invited to take part in the study by poster advertisements and survey invitations by e-mail and phone calls. All volunteers that agreed to take part in the clinical trial signed the informed consent and received full explanation of the purposes and procedures of the study. Eligibility of candidates to enter the study was performed according to a screening process that included medical history, physical examination, recording of anthropometric measurements and blood samples for evaluation of hematology tests, glycated hemoglobin (HbA1c), glucose, insulin, liver function tests, blood chemistry, insulin resistance by the homeostatic model assessment (HOMA) and Human Chorionic Gonadotropin Hormone (in women). HOMA was calculated as follows: (Fasting Plasma Insulin) x (Fasting Plasma Glucose/22.5). Subjects enrolled in the study met the cutoff point of 3.8, as described by Qu et al [20]. Eligibility criteria also included the acceptance of not consuming any foods or beverages containing NNS according to a list dispensed, attending the weekly appointments, and refraining from smoking and alcohol ingestion throughout the study. Women were enrolled in the study if they used at least one method of contraception in order to prevent pregnancy.

Participants with previous diagnosis of any acute or chronic disease, malabsorption syndrome or short bowel syndrome, use of corticosteroids, antibiotics or non-steroidal anti-inflammatory drugs (NSAID) during the last 3 months prior to enter the study were excluded. Once enrolled, participants that attended less than 80% of weekly appointments, fell pregnant, smoked cigarettes or consumed alcohol were excluded from the study.

### Sample size

The sample size estimation was performed using the GPower v.3.1 9.2 program [21], expecting an effect size of 0.14, with an alpha error of 0.05 and a power of 95%, for three groups and 11 repeated measures that resulted in a sample size of 44 participants per group.

### Randomization and blinding

BHN randomized participants into three groups, as follows: a) subjects receiving 48 mg sucralose, b) subjects receiving 96 mg sucralose, and c) subjects receiving water as placebo. Each group ingested sucralose or water as placebo every day, for 10 weeks. The allocation groups were unrevealed to the participants as well as to researchers who delivered sucralose or placebo, or to who conducted the weekly follow-ups. Randomization was generated using the Web site http://www.randomization.com including 6 individuals per block.

### Sucralose intervention

Sucralose solution (20% w/v, Tate & Lyle Decatur, IL, USA) was diluted with pure drinking water. Clean, sterile, dark plastic bottles were filled with 60 mL of diluted sucralose, so that each bottle contained 48 mg (2 mM) or 96 mg (4 mM) sucralose. Placebo was prepared by just filling the bottles with 60 mL of water. In all cases, the liquids were not visible, colorless and not flavored. Cardboard boxes were assembled, each one containing 9 bottles with the same amount of sucralose or water. RBA prepared and placed the beverages in the bottles. BHN labeled the cardboard boxes according to the randomization scheme. ASR disseminated the beverages to participants. Each volunteer received one box every week.

In order to evaluate energy intake (Kcal/d) and diet composition (g/d of lipids, proteins and carbohydrates), food frequency questionnaires from the past 7 days were applied in each weekly visit and intolerance symptoms or adverse events, if any, were registered. To evaluate participant dietary intake, researchers (GAAY, ASR, EFAJ, LBKL and MMVM) were trained and administered 24-h food recalls and the Food Frequency Questionnaire with Intense Sweeteners (FFQIS), previously validated by our group [22]. To assess compliance, the empty bottles in each box were returned during weekly visits and a new box containing filled-up bottles was dispensed.

### Clinical evaluation of volunteers

The participants’ total cholesterol, high-density lipoproteins (HDL), low-density lipoproteins (LDL), triglycerides, waist-to-hip ratio (WHR) and NNS consumption (mg/d) were measured before starting the intervention.

At week zero (Wk0) and at week 10 (Wk10), bioimpedance and anthropometry assessments were performed using RJL Quantum IV system (RJL Systems Inc. Clinton Township, MI. 48,035, USA), a scale and a metric tape. An Oral Glucose Tolerance Test (OGTT), HbAc1, fasting glucose and fasting insulin were assessed at Wk0 and Wk10.

The Matsuda insulin sensitivity index was calculated for control and sucralose groups at Wk0 and Wk10, as described elsewhere [23].

Researchers (RBA and LHM) obtained blood samples. The biochemical analysis of blood samples was performed by trained personnel of the Hospital’s Central Laboratory. Researchers (GAAY, ASR, EFAJ, LBKL and MMVM) collected anthropometrical and bioimpedance measurements from the participants and calculated WHR and Matsuda index.

### Oral Glucose Tolerance Tests

OGTTs were performed in participants with 8–10 h fasting. Briefly, a cannula was introduced in the antecubital vein. Fifteen minutes before the glucose load (75 g in 240 mL of water), one dose (one bottle) of sucralose or placebo was drank by each participant. Blood samples were drawn at − 15 min (immediately before drinking sucralose or placebo), at 0 min (immediately before drinking the glucose load), and at 15, 30, 45, 60, 75, 90, 105, 120 and 180 min, consecutively. Blood samples were sent to the hospital’s central lab for immediate glucose and insulin measurements. At the end of the OGTT, all participants received a light lunch. Plasma glucose was measured using a glucose analyzer (Beckman Coulter, glucose, CA, USA) and plasma insulin was measured through an enzyme-linked immunosorbent assay (ELISA, Beckman Coulter, Ultrasensitive insulin, CA, USA).

### Statistical analysis

Data entry was performed twice by two separate managers. Demographic data were reported as means and standard deviations or proportions. The Kolmogorov-Smirnov test was used to assess normality for each variable.

Areas under the curve (AUC), maximal concentration and time to maximal concentration from Wk0 and Wk10 of the OGTT, were also calculated for each group by the trapezoid method, using the pk module of the Stata 15 software (StataCorp LLC, College Station, TX, USA).

Differences between Wk0 and Wk10 of each group were evaluated by two-tailed paired-t tests. Differences between all groups at the same time point (Wk0 or Wk10) were compared by One-Way ANOVA. Differences between two groups at the same time points were analyzed using two-tailed t-tests.

Univariant Analysis of Variance was performed to determine the existence of interactions between interventions (sucralose and placebo) and time (Wk0 and Wk10). A two-way ANOVA was performed to analyze differences between Wk0 and Wk10, using the Bonferroni test to adjust for multiple comparisons.

The IBM-SPSS version 23 statistical package (Chicago, Ill, USA) was used for the analysis. A *p* value < 0.05 was set as statistically significant and p between 0.05 and 0.1 was considered to show a tendency.

## Results

**Fig. 1:**
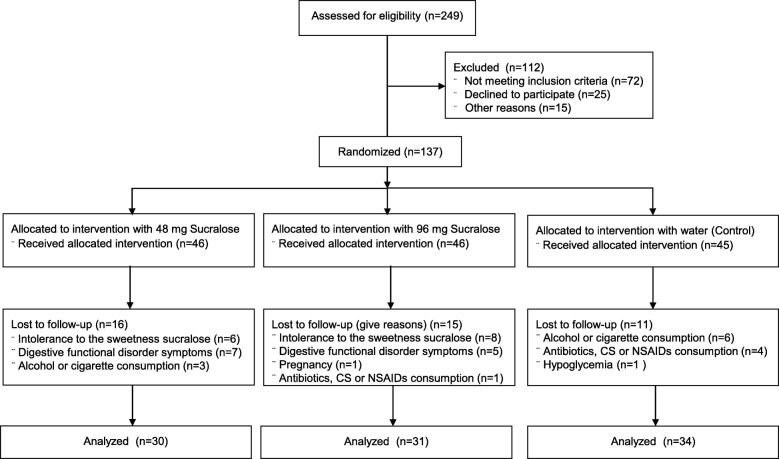
Flow chart of the study. The flow diagram shows the progress of the participants who were recruited, randomized and followed up throughout the study, according to the CONSORT (Consolidated Standards of Reporting Trials) guidelines

Two hundred forty nine young adults were invited to the study (from February 2016 to June 2019); 137 accepted to participate and met the inclusion and exclusion criteria to enter the study. After randomization, assignments were 45 subjects in the control group (placebo), 46 subjects in the 48 mg sucralose group, and 46 subjects in the 96 mg sucralose group. By the end of Wk10, only 95 participants completed the study; 34 in the control group, 30 in the 48 mg sucralose group and 31 in the 96 mg sucralose group, and these were considered for statistical analyses. The principal reasons for withdrawal were intolerance to the sweetness of sucralose and digestive functional disorders (Fig. 1).

Baseline characteristics in all three groups were similar, including age, HOMA-IR, HbAc1, BMI, WHR, body composition (body fat, fat free mass, lean dry mass and total body water), total cholesterol, HDL, LDL and triglycerides. Women were more prevalent than men in the three study groups (Table 1).

**Table 1:** Baseline characteristics of the volunteers

	48 mg ***n*** = 30	96 mg ***n*** = 31	Control ***n*** = 34	p^**a**^
Age, years (±SD)	22.9 ± 3.5	22.6 ± 2.8	22 ± 3.2	0.86
Gender, n				
Females	20	18	20	0.75
Males	10	13	14	0.43
BMI, kg/m^2^	24.1 ± 2.9	23.8 ± 3.3	24.2 ± 3.8	0.84
WHR, cm/cm	0.8 ± 0.07	0.8 ± 0.08	0.81 ± 0.07	0.69
Body fat, %	37.2 ± 5.2	34.4 ± 5.9	35.5 ± 7.2	0.21
Fat free mass, kg	39.9 ± 8.2	43.1 ± 9.2	41.6 ± 8.5	0.34
Lean dry mass, kg	11.3 ± 2.03	12.04 ± 2.1	11.6 ± 2.02	0.42
Total body water, %	44.8 ± 4.3	47.09 ± 4.5	46.2 ± 5.4	0.19
Total cholesterol, mg/dL	163.4 ± 28.3	165.9 ± 33.5	161.5 ± 27.9	0.83
HDL cholesterol, mg/dL	41.6 ± 9.6	45.2 ± 10.3	43.09 ± 9.5	0.34
LDL cholesterol, mg/dL	95.5 ± 22.5	98.2 ± 29.7	97.2 ± 24.8	0.91
Triglycerides, mg/dL	125.5 ± 110.5	102.5 ± 69.7	99.8 ± 58.3	0.39
HOMA-IR	1.71 ± 0.7	1.48 ± 0.7	1.61 ± 0.6	0.41
HbAc1, mg/dL (±SD)	5.2 ± 0.2	5.1 ± 0.3	5.2 ± 0.2	0.38
Intake				
Energy, Kcal/d	1935.9 ± 681.7	2133. ± 965.8	2264.1 ± 1247	0.42
Lipids, g/d	67.6 ± 43.4	69.2 ± 43.3	71.5 ± 59.9	0.95
Protein, g/d	94.4 ± 60.6	100.7 ± 44.3	103.9 ± 74.8	0.82
Carbohydrate, g/d	227.8 ± 59.32	264.7 ± 133.9	289.4 ± 118.4	0.08
NNS, mg/d	102 ± 287.5	68 ± 140.3	74.34 ± 141.5	0.84
Consumption of NNS n (%)	21 (70)	20 (64)	23 (73)	0.56
Adherence to intervention (%)	94.14 ± 11.81	86.07 ± 19.12	87 ± 20.37	0.16

*BMI* body mass index, *HOMA* hemoglobin model assessment, *Hb1Ac* hemoglobin glycated, *HDL* high density lipoproteins, *LDL* low density lipoproteins, *SD* standard deviation, *WHR* waist-to-hip ratio, *g/d* grams per day ^a^ One-Way ANOVA

### Chronic consumption of sucralose increases insulin responses in OGTT

**Fig. 2:**
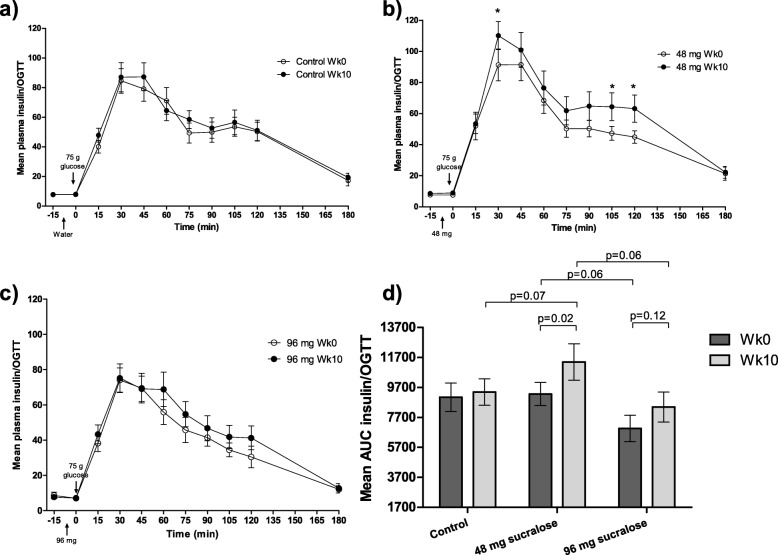
OGTT insulin concentration values of the participants. Wk0 and Wk10 OGTT insulin curves are depicted for **a**) control group, **b**) 48 mg sucralose group and c) 96 mg sucralose group. d) Mean insulin AUC compared between Wk0 and Wk10 OGTTs for each group. Arrows indicate the time in which sucralose or water (placebo) were administered, as well as the glucose load. Data are mean ± SEM. *p < 0.05. Statistical analysis: Two-tailed t-tests and two-tailed paired-t tests

**Fig. 3:**
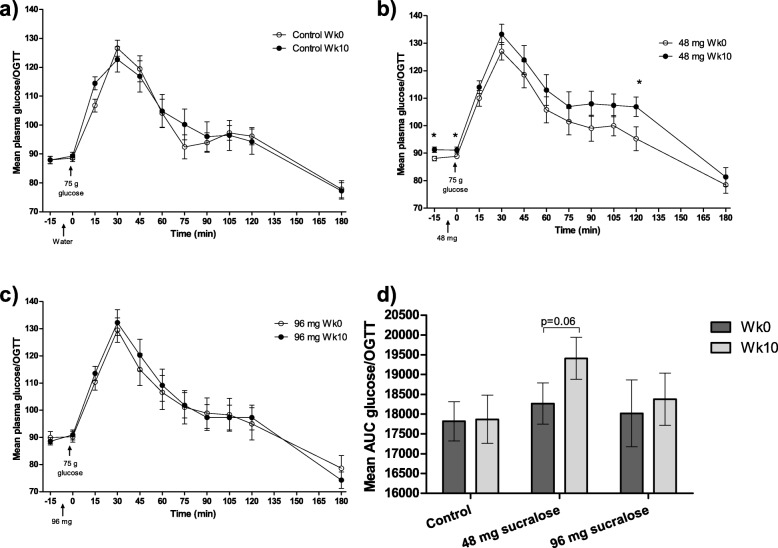
OGTT glucose concentration values of the participants. Wk0 and Wk10 OGTT glucose curves are depicted for **a**) control, **b**) 48 mg sucralose and **c**) 96 mg sucralose. d) Mean glucose AUC compared between Wk0 and Wk10 OGTTs for each group. Arrows indicate the time in which sucralose or water (placebo) were administered, as well as the glucose load. Data are mean ± SEM. *p < 0.05. Statistical analysis: two-tailed paired-t tests

In order to identify if the consumption of sucralose for 10 weeks (chronic effect) might provoke derangements in the carbohydrate metabolism, we compared glucose and insulin levels in OGTTs at Wk0 and at Wk10. In the control group, no differences were found in insulin and glucose concentrations between Wk0 and Wk10 (Fig. 2d and Fig. 3d). In the 48 mg sucralose group, significant increases in insulin were found at 0 min (7.5 ± 3.4 μIU/mL vs 8.8 ± 4.1 μIU/mL; *p* = 0.01), at 30 min (91.3 ± 56.2 μIU/mL vs. 110.1 ± 49.4 μIU/mL; *p* = 0.05), at 105 min (47.7 ± 24.4 μIU/mL vs. 64.3 ± 48.2 μIU/mL; *p* = 0.04), and at 120 min (44.8 ± 22.1 μIU/mL vs. 63.1 ± 47.8 μIU/mL; p = 0.01) (Fig. 2b). In the same group, a significant increase in glucose was found at − 15 min (87.9 ± 4.6 mg/dL vs 91.4 ± 5.4 mg/dL; *p* = 0.003), at 0 min (88.7 ± 4 mg/dL vs. 91.3 ± 6 mg/dL; p = 0.04), and at 120 min (95.2 ± 23.7 mg/dL vs 106.9 ± 19.5 mg/dL; p = 0.01). Although glucose concentration at Wk10 was higher than that found at Wk0, differences were not significant (Fig. 3b). In the 96 mg sucralose group, insulin and glucose concentrations increased but none reached significant differences (Fig. 2c and Fig. 3c).

To compare the global response to insulin during OGTTs, we performed insulin and glucose AUC analyses. First, we analyzed if there were interactions between type of intervention and consumption time. No interaction between type of intervention (control, 48 mg or 96 mg sucralose) and consumption time (Wk0 and Wk10) was found in glucose AUC (F(2,184) = 0.00, *p* = 1.00). Consumption time had no effect on glucose AUC (F(1,184) = 0.00, p = 1.00); in contrast, type of intervention showed a significant effect (F(2,184) = 3.327, *p* = 0.038, η^2^ = 0.035, 1-β = 0.624). A similar trend was found in the AUC of insulin, where no interaction between type of intervention and consumption time was observed (F(2,184) = 0.45, *p* = 0.63). Consumption time did not have a significant effect on the insulin AUC, however, it showed a tendency to increase (F(1,184) = 2.77, *p* = 0.10). In contrast, type of intervention had a significant effect on the AUC of insulin (F(2,184) = 3.74, p = 0.03, η^2^ = 0.039, 1-β = 0.679). Two-way ANOVA analysis showed no interaction between type of intervention and consumption time in both glucose and insulin AUCs (F(4,366) = 0.255, *p* = 0.91). However, type of intervention showed a significant effect on (a) the insulin AUC when compared subjects of the Bonferroni’s correction showed that this effect was significant for insulin AUC between 48 mg sucralose group versus participants of the 96 mg sucralose group (means difference: − 2652.203, SE: 975.45, CI 95%: − 5008.957, − 295. 449; *p* = 0.02), and (b) glucose AUC when compared volunteers of the 48 mg sucralose group versus control subjects (means difference: -1537.81, SE: 603.475, CI 95%: − 2995.848, − 79.772; *p* = 0.035) (Table 2).

**Table 2:** Differences between experimental groups (48 mg and 96 mg sucralose) and control group at Wk0 and Wk10 OGTT

	(I) Group	(J) Group	Media difference (I-J)	Standard error	p	95% Confidence interval	
						Lower Bound	Upper Bound
Glucose, AUC	Control	48 mg	-1537.81	603.48	0.03^a^	-2995.85	−79.77
		96 mg	− 509.85	598.28	1.00	− 1955.34	935.64
	48 mg	Control	1537.81	603.48	0.03^a^	79.77	2995.85
		96 mg	1027.96	617.01	0.29	−462.79	2518.70
	96 mg	Control	509.85	598.28	1.00	−935.64	1955.34
		48 mg	− 1027.96	617.01	0.29	− 2518.70	462.79
Insulin, AUC	Control	48 mg	− 1104.65	954.05	0.75	− 3409.70	1200.40
		96 mg	1547.55	945.84	0.31	− 737.66	3832.77
	48 mg	Control	1104.65	954.05	0.75	− 1200.40	3409.70
		96 mg	2652.20^a^	975.45	0.02	295.45	5008.96
	96 mg	Control	− 1547.55	945.84	0.31	− 3832.77	737.66
		48 mg	−2652.20^a^	975.45	0.02	−5008.96	−295.45

^a^ The mean difference is significant at 0.05 level. Two-way ANOVA adjustment for multiple comparisons with Bonferroni

We then compared both glucose and insulin AUCs at Wk0 and at Wk10. Insulin AUC showed the most significant increases after 10 weeks of exposure to 48 mg sucralose (AUC Wk0 = 9262 ± 4225 vs AUC Wk10 = 11,398 ± 6641; p = 0.02) but not to 96 mg sucralose (AUC Wk0 = 6962 ± 4899 vs AUC Wk10 = 8394 ± 5567; *p* = 0.12) (Fig. 2d). Insulin AUC showed no significant differences between Wk0 and Wk10. However, insulin AUC tended to increase in the 48 mg sucralose group with respect to that found in the control group at Wk10 (AUC Wk10 48 mg = 11,398 ± 6641 vs AUC Wk10 control = 9397 ± 5126; *p* = 0.07). At Wk0, insulin AUC tended to decrease in the 96 mg sucralose group as compared to that found in the 48 mg sucralose group (AUC Wk0 96 mg = 6962 ± 4899 vs AUC Wk0 48 mg = 9262 ± 4225; *p* = 0.06). At Wk10, insulin AUC tended to decrease in the 96 mg sucralose group as compared to that found in the 48 mg sucralose group (AUC Wk10 96 mg = 8394 ± 5567 vs AUC Wk10 48 mg = 11,398 ± 6641 p = 0.06) (Fig. 2d).

Similarly, glucose AUC showed no significant changes after 10 weeks of drinking 48 mg sucralose; however, a tendency to increase was seen (AUC Wk0 = 18,267 ± 2858 vs AUC Wk10 = 19,408 ± 2904; p = 0.06) (Fig. 3d).

**Fig. 4:**
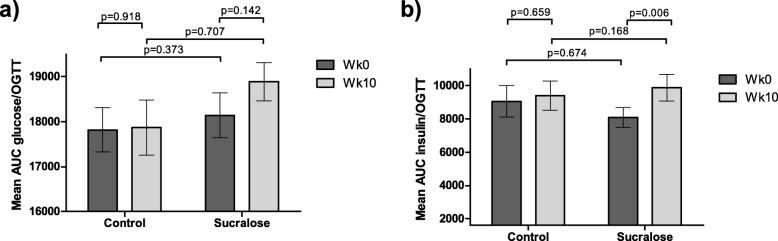
Analysis of Wk0 and Wk10 glucose and insulin AUC between the sucralose exposed group (48 mg & 96 mg groups) and not exposed group (control). Data are mean ± SEM. *p < 0.05. Statistical analysis: Two-tailed paired-t tests

We then analyzed glucose AUCs between subjects receiving any sucralose amount (either 48 or 96 mg sucralose) and those drinking placebo. After 10 weeks of exposure, we found that volunteers receiving any sucralose concentration had higher glucose and insulin levels than those found in controls receiving placebo (for glucose, Wk0 = 18,141 ± 3877 vs Wk10 = 18,885 ± 3332, *p* = 0.01; for insulin, Wk0 = 8093 ± 4687 vs Wk10 = 9871 ± 6253, *p* = 0.006). It is worth mentioning that both glucose and insulin AUCs were similar when compared Wk0 versus Wk10 in the control group (Fig. 4).

Furthermore, the maximum insulin peak significantly increased in the 48 mg sucralose group after 10 weeks of intervention (Max [Ins] Wk0 = 106.1 ± 57.9 vs Max [Ins] Wk10 = 130.9 ± 60.51, p = 0.01) (Table 3).

**Table 3:** Change on metabolic parameters and macronutrients consumption in the diet

	Wk0	Wk10	p^**a**^
Insulin, AUC			
48 mg sucralose	9262 ± 4225	11,398 ± 6641	0.02
96 mg sucralose	6962 ± 4899	8394 ± 5567	0.12
Control	9054 ± 5571	9397 ± 5126	0.65
p^b^	0.135	0.125	
Glucose, AUC			
48 mg sucralose	18,267 ± 2858	19,408 ± 2904	0.05
96 mg sucralose	18,019 ± 4705	18,380 ± 3677	0.66
Control	17,820 ± 2872	17,870 ± 3557	0.91
p^b^	0.883	0.195	
Matsuda			
48 mg sucralose	6.04 ± 3.19	4.86 ± 2.13	0.01
96 mg sucralose	7.93 ± 4.63	7.09 ± 4.72	0.30
Control	6.8 ± 3.35	6.14 ± 3.41	0.09
p^b^	0.14	0.05	
Max [Glu]			
48 mg sucralose	133.06 ± 20.79	141.17 ± 18.59	0.09
96 mg sucralose	133.45 ± 29.72	137.61 ± 25.33	0.46
Control	131.94 ± 21.19	135.12 ± 23.53	0.41
p^b^	0.96	0.56	
Max [Ins]			
48 mg sucralose	106.10 ± 58	130.9 ± 60.51	0.01
96 mg sucralose	85.42 ± 42.67	90.42 ± 50.41	0.48
Control	100 ± 56.07	108.94 ± 62.15	0.34
p^b^	0.29	0.02	
Time to max [Glu]			
48 mg sucralose	34 ± 18.45	40.5 ± 23.35	0.11
96 mg sucralose	36.77 ± 20.06	34.84 ± 19.51	0.58
Control	36.18 ± 9.85	36.18 ± 20.27	1.00
p^b^	0.78	0.55	
Time to Max [Ins]			
48 mg sucralose	40.5 ± 14.82	38.5 ± 20.35	0.58
96 mg sucralose	40.65 ± 17.4	45.48 ± 17.53	0.23
Control	45 ± 19.19	48.09 ± 23.87	0.50
p^b^	0.49	0.17	
BMI, kg/m^2^			
48 mg sucralose	24.2 ± 2.9	24.2 ± 3.1	0.47
96 mg sucralose	23.7 ± 3.3	23.6 ± 3.2	0.56
Control	24.2 ± 3.8	24.1 ± 3.9	0.32
p^b^	0.84	0.76	
Carbohydrates, g/d			
48 mg sucralose	227.9 ± 59.3	252.9 ± 86.7	0.18
96 mg sucralose	264.7 ± 133.9	286.5 ± 132.7	0.42
Control	289.4 ± 118.4	250.7 ± 123.5	0.12
p^b^	0.08	0.42	
Lipids, g/d			
48 mg sucralose	67.6 ± 43.4	81.6 ± 40.4	0.10
96 mg sucralose	69.2 ± 43.4	74.2 ± 40.9	0.63
Control	71.6 ± 59.9	62.1 ± 26.6	0.29
p^b^	0.95	0.11	
Proteins, g/d			
48 mg sucralose	94.4 ± 60.6	97.47 ± 44.4	0.86
96 mg sucralose	100.8 ± 44.4	94.9 ± 82.9	0.32
Control	103.9 ± 74.8	84.7 ± 32.9	0.09
p^b^	0.824	0.427	
Energy, Kcal/d			
48 mg sucralose	1935.9 ± 681.8	2170.7 ± 732.3	0.10
96 mg sucralose	2133.1 ± 965.8	2235.7 ± 917.5	0.65
Control	22 64.8 ± 1247.2	1941.6 ± 710.7	0.09
p^b^	0.42	0.30	
HbA1c			
48 mg sucralose	5.23 ± 0.21	5.26 ± 0.26	0.51
96 mg sucralose	5.15 ± 0.3	5.25 ± 0.26	0.03
Control	5.22 ± 0.23	5.24 ± 0.24	0.55
p^b^	0.38	0.94	

*AUC* area under the curve, *BMI* body mass index, *Hb1Ac* hemoglobin glycated, *HOMA* homeostatic model assessment, *Wk0* week 0, *Wk10* week 10, 48 mg (n = 30); 96 mg (n = 31); Control (n = 34). Media ± standard deviation

^a^ Two-tailed paired-t tests; ^b^ Differences between groups, One-Way ANOVA

### Sucralose chronic consumption modifies insulin sensitivity

The Matsuda index for insulin sensitivity was calculated at Wk0 and Wk10 for each group. After 10 weeks, the Matsuda index showed a significant reduction in the 48 mg sucralose group (Wk0 = 6.04 ± 3.19 vs Wk10 = 4.86 ± 2.13; p = 0.01), but not in the 96 mg sucralose group (Wk0 = 7.93 ± 4.6, Wk10 = 7.09 ± 4.71, *p* = 0.30) (Table 3).

Overall, food consumption showed no significant differences among the three intervention groups in carbohydrates, lipids, proteins, and energy intake (Table 3). After 10 weeks, the average carbohydrate intake decreased only in control subjects (Wk0 = 289.4 ± 118.4 vs Wk10 = 250.7 ± 123.5; *p* = 0.12), whereas in volunteers receiving sucralose increased (Wk0 48 mg = 227.9 ± 59.3 vs Wk10 48 mg = 252.9 ± 86.7; *p* = 0.18; Wk0 96 mg = 264.7 ± 133.9 vs Wk10 96 mg = 286.5 ± 132.7; *p* = 0.42). However, no significant differences were reached.

Adherence to intervention was also similar in the three groups; however, the highest adherence rate was found in the 48 mg sucralose group (94%) while subjects in the 96 mg sucralose group showed the lowest adherence rate (86%).

### Acute consumption of sucralose has little to no impact in the insulin response during OGTT

The effect of the acute consumption of sucralose was assessed by comparing insulin values at − 15 min and at 0 min of the OGTTs performed at Wk0. No differences were found between placebo and sucralose groups (48 mg or 96 mg), (Figs. 2 and 3).

### Adverse events

Two volunteers developed symptoms of hypoglycemia during OGTTs, one of the control group and the other one of the 96 mg sucralose group. However, these symptoms were mild and volunteers recovered soon. Volunteers also reported several digestive functional disorders (Fig. 1).

## Discussion

The most notable findings observed in the study were changes in insulin concentrations, insulin AUCs, and glucose AUCs in the 48 mg sucralose intervention group at 10th week. The most significant changes in insulin were found at 0, 30, 105 and 120 min, possibly as a delayed metabolic response generated to compensate the insulin increment to keep glucose on target. Likewise, the effect on the AUC was greater in insulin than in glucose. This data suggest that chronic consumption of sucralose reduces insulin sensitivity in young adults; in consequence, insulin production tended to increase with the aim of keeping glucose levels in normal values. Insulin did not only change, also a reduction in the Matsuda Index was found in the same group, which in turn supports the idea that insulin exerted a compensatory response to chronic sucralose consumption.

Unexpectedly, these effects were only significant in the 48 mg sucralose group but not in the 96 mg sucralose group; possible explanations are discussed later.

One unexpected finding was the high variability in OGTT glucose and insulin time points among participants; we consider that this resulted from the high HOMA value that we decided to set as cut off point (< 3.8).

Several studies have shown that sucralose is not physiologically innocuous. Pepino et al, reported that acute consumption of 48 mg sucralose increased glucose concentrations as well as insulin AUC in a 5 h OGTT in individuals with obesity [10]. However, this work was criticized due to the small number of volunteers enrolled, mostly women [24]. We did not find any differences attributable to the acute consumption of sucralose, however, we found differences attributable to its chronic consumption: maximum insulin concentration (or peak insulin) was increased in the 48 mg sucralose group and two OGTT insulin time points showed reduction in the 96 mg sucralose group as compared to placebo (105 min and 120 min). Nonetheless, Pepino’s data in obese patients resemble those here reported in young healthy volunteers with normal BMI, which suggests that there is induced reduction in insulin sensitivity associated with sucralose consumption for 10 weeks. Also, Sylvetsky et al observed an increase in insulin levels (peak insulin and AUC) but not glucose, during a two-hour OGTT in healthy adults who received one diet soda containing sucralose and acesulfame K prior to the glucose load. This effect was also observed when volunteers received sucralose and acesulfame K in seltzer water, but it was not statistically significant [25].

Furthermore, Lertrit et al reported in a randomized double-blind cross over study that daily oral ingestion of 200 mg encapsulated sucralose for 4 weeks decreased whole body and hepatic insulin sensitivities (Matsuda index and HOMA-IR, respectively) in 15 healthy volunteers without obesity (11 female). Sucralose was administered in capsules, which avoided the oral response to the sweetness [26]. Nonetheless, the effect that both Lertrit et al and we found in insulin sensitivity suggests that sucralose may reduce insulin sensitivity through extraoral responses.

On the contrary, the 12-week study in 46 normoglycemic male volunteers who received ~ 333.3 mg encapsulated sucralose or placebo 3x/day test, showed no significant differences between Wk0 and Wk10 OGTTs in HBA1c, glucose, insulin, and C peptide [27]. In light of this information, our results support the hypothesis that continuous consumption of sucralose affects insulin release to take glucose into the organs. The individuals in our study, healthy young adults with no previous carbohydrate metabolism impairment, were challenged with a dose equivalent to one (48 mg) or two cans (96 mg) of regular diet beverages, an amount that is easily reachable in modern diet.

We also found a statistically significant increase in HbA1c after consumption for 10 weeks of 96 mg sucralose. Davis et al also reported an increase in HbA1c in Hispanic young individuals with overweight or obesity who were frequent consumers of beverages with NNS at least for 1 year [28]. This effect could also depend on the diet content, for instance, sucralose consumption (≥5 days) in combination with an imbalanced diet has been shown to trigger a neuronal response which stimulates food intake in a fruit fly model [29]; nevertheless, in the present study no significant differences were found in the intake of macronutrients in any of the groups before and after the intervention for 10 weeks. In view of these results, it seems that sucralose has metabolic effects that interfere with glucose and insulin homeostasis, possibly due to different mechanisms such as gut microbiota dysbiosis or sweet taste receptors expressed in the digestive system [30–32], which play an important role in both glucose absorption and insulin secretion.

It is pertinent to mention that the effects of sucralose consumption for 10 weeks on insulin and glucose were only observed in the 48 mg sucralose group and not in the 96 mg sucralose group. Several explanations are possible that can be partially involved in this phenomenon. For instance, lower adherence to intervention in the 96 mg sucralose group than in the 48 mg group; however, this difference was not so great to completely explain what was observed. Likewise, the excessive sweetness of this dose of sucralose may have provoked an under consumption of the NSS that was not reported by participants. Other possible explanations may involve different mechanisms related to glucose and insulin homeostasis, like gut microbiota dysbiosis, sweet taste receptors response, cephalic response to sucralose, among others, that are discussed below.

Sucralose induces a cephalic phase in the insulin response without any other stimulus, which can influence digestion and metabolism leading to the release of several hormones such as GLP-1, ghrelin, and insulin after oral exposure [17]. In our sample, we could not determine the cephalic phase of the insulin response, a research question that will be answered further.

Likewise, gut microbiota could also play a role in explaining the differences between groups. In this sense, Suez et al reported impaired glucose tolerance in mice after exposure to saccharin, sucralose or aspartame in the drinking water for 10 weeks, an effect that was dependent on the alteration in gut microbiota caused by NNS [11]. Moreover, they reported a significant correlation between NNS consumption and metabolic syndrome in 381 individuals without diabetes (56% females); and significantly poorer glycemic responses 5–7 days after saccharin consumption for 1 week in four out of seven healthy volunteers who were exposed to NNS for 7 days; in both cases, alterations in the microbiota composition (dysbiosis) were present in those individuals exposed to NNS [11]. This data highlight the importance of gut microbiota in glucose and insulin homeostasis; also, they show the great variability in microbiota among individuals, and how it affects glycemic responses, as not all individuals exposed to NNS featured the same outcomes. In our study we did not assessed changes in gut microbiota related to sucralose consumption; it is possible that more individuals with dysbiosis are present in either the 96 mg sucralose group than in the 48 mg sucralose group, which can bias the glucose and insulin responses during OGTT, especially considering that the amount of NNS consumed between these two groups tended to differ.

Other mechanisms that may also explain the response observed in the 96 mg sucralose group can be related to sweet receptor signaling in cells. This receptor, a TAS1R2/TAS1R3 heterodimer, is expressed in several tissues, from the upper gastrointestinal tract to intestinal brush and enteroendocrine cells [18] and recognizes natural and NNS. It has been reported that sucralose, as well as sucrose and glucose, induces the release of GLP-1 in human enteroendocrine cells NCI-H716, nevertheless, at higher concentrations (20 mM), GLP-1 release is inhibited and returns to basal levels [33], which implies that there is a not-yet described mechanism that quenches the signal. In the present study, none of the intervention groups received such a high dose (48 mg and 96 mg of sucralose are equivalent to 2 mM and 4 mM, respectively); however, sucralose pharmacokinetics in the human body should be considered. Sucralose takes more than 72 h to be eliminated; in feces and urine sucralose can be detected more than 72–96 h after ingestion of a single dose, while in plasma sucralose is still detectable 72 h after ingestion [34]. Since we gave a daily dose of sucralose for 10 weeks to participants, sucralose may have been accumulated thus increasing the total amount to which participants were exposed, blunting the signals of sucralose on the receptors, especially in the 96 mg sucralose group.

Besides, it should be mentioned that our study did not find a beneficial effect of sucralose consumption on BMI, HOMA, cholesterol or triglycerides, which agrees with other studies that reported limited or no effects of NNS consumption on reduction of caloric intake and consequently BMI or other clinical manifestations associated with obesity [35]. Even more, in adolescents the consumption of NNS has been associated with greater probability of obesity [36]. To our knowledge, this is the largest randomized, double blind, controlled trial challenging the glucose-insulin axis in healthy volunteers, with normal weight and HOMA values, who were exposed to NNS with two different sucralose amounts (48 and 96 mg) for 10 weeks.

### Limitations of the study

A major limitation in our study was the limited effects in the 96 mg sucralose group, since the exact mechanism of the observed effects is unknown. Differences in adherence to intervention between groups also limit data interpretation. Furthermore, the moment at which sucralose was ingested daily was not registered, therefore subjects may not have ingested the sucralose at the same hour daily, or may have combined it with different foods, which may have altered the response to intervention. Nonetheless, in a real scenario, people do not ingest sucralose alone diluted in simple water; sucralose is found in different foods and beverages which contain a complex mixture of ingredients and often, other NNS too. Also, diet was not controlled, however, there were no statistically significant differences in carbohydrate, lipids or proteins intake between groups after 10 weeks of sucralose or placebo ingestion, although micronutrients were not measure. Likewise, gut microbiota changes were not assessed.

Finally, the use of water as a placebo in the control group may not be the best election in all studies, especially those who wish to assess the cephalic response to sweetness. In our case, we did not avoid this response, however, if we had used another substance as a control, for example, another NNS or sugar, we would not have discerned the effect of sucralose from that of the substance used as control. Further studies with sucralose encapsulated should be still conducted.

## Conclusion

This study adds new evidence regarding the effect of chronic sucralose consumption on serum insulin and insulin sensitivity.

An effect of chronic consumption of sucralose on insulin response in healthy volunteers subjected to an OGTT was observed, however, the effect was not consistent with dose.

Further research is required before mayor claims can be made regarding the effect of sucralose consumption on glucose and insulin responses.

## Data Availability

We have made publicly and freely available without restriction the data described in the manuscript, at: https://www.dropbox.com/s/8waa4iev50eeoo9/Public%20database.xlsx?dl=0

## Abbreviations

ANOVA: Analysis of variance
AUC: Areas under the curve
BMI: Body mass index
GLP − 1: Glucagon-like peptide 1
Hb1Ac: Hemoglobin glycated
HDL: High density lipoproteins
HOMA: Hemoglobin model assessment
LDL: Low density lipoproteins
NNS: Non-nutritive sweeteners
NSAID: No steroidal anti-inflammatory drugs
OGTT: Oral glucose tolerance test
SD: Standard deviation
TAS1R2: Taste receptor type 1 member 2
TAS1R3: Taste receptor type 1 member 3
WHR: Waist-to-hip ratio
